# Fatal Outcome of European Tick-borne Encephalitis after Vaccine Failure

**DOI:** 10.3389/fneur.2017.00119

**Published:** 2017-04-03

**Authors:** Parham Sendi, Cédric Hirzel, Stefan Pfister, Rahel Ackermann-Gäumann, Denis Grandgirard, Ekkehard Hewer, Arto C. Nirkko

**Affiliations:** ^1^Institute for Infectious Diseases, University of Bern, Bern, Switzerland; ^2^Bern University Hospital, Department of Infectious Diseases, Bern, Switzerland; ^3^Spiez Laboratory, Swiss National Reference Centre for Tick-transmitted Diseases, Federal Office for Civil Protection, Spiez, Switzerland; ^4^Institute of Pathology, University of Bern, Bern, Switzerland; ^5^Department of Neurology, Schlaf-Wach-Epilepsie-Zentrum (SWEZ), University of Bern, Bern, Switzerland

**Keywords:** tick-borne encephalitis, tick-borne encephalitis vaccine, vaccine efficacy, vaccine failure, flavivirus

## Abstract

Tick-borne encephalitis is a viral disease affecting the central nervous system. It is endemic in Switzerland with 200–250 notified cases annually. Active immunization is effective for persons in all age groups. Vaccine failure is rare, in particular after a completed vaccination course. Here, we describe the case of 67-year-old man with a fatal outcome despite vaccination. The diagnosis was confirmed by extensive postmortem analyses. The diagnostic challenges of vaccine failure in tick-borne encephalitis and the dynamics of the immune response in vaccination breakthrough are discussed.

## Introduction

Tick-borne encephalitis virus (TBEV) can cause severe meningoencephalitis with substantial morbidity. The vaccine against TBEV has shown high efficacy (1). Vaccine failures—as presented in this case with a fatal outcome—are rare, in particular when the vaccine series has been completed. Vaccine failures often cause diagnostic challenges and hence are associated with delayed diagnosis. The clinical, electrophysiological, and neuroimaging evolution of this case, which included serial serum and cerebrospinal fluid (CSF) samples and brain biopsy, together with postmortem analyses, may yield insights into the dynamics of the immune response in TBE vaccination breakthrough.

## Case Report

The clinical diagnosis of meningitis was raised in a 67-year-old farmer when he presented with headaches, fever, nausea, and vomiting. The symptoms started 1 day prior to presentation. He was treated intravenously with ceftriaxone, amoxicillin, and acyclovir. His laboratory studies revealed an elevated white blood cell (WBC) count of 15,600/μL with 4.5% bands. His C-reactive protein level was 19.2 mg/L, and his erythrocyte sedimentation rate was 13 mm/h. Blood and urine culture yielded no growth of microorganisms. A computed tomography (CT) scan of the head showed no pathologic findings. CSF analysis revealed a WBC count of 244/μL (46/μL neutrophils and 198/μL lymphocytes), a protein level of 0.38 g/L and a glucose level of 3.86 mmol/L (in comparison to glucose in serum of 6.51 mmol/L and a CSF-serum ratio of 0.6). The results of a CSF culture for bacteria, fungi, and mycobacteria were negative. Polymerase chain reaction (PCR) analyses for herpes simplex virus (HSV) 1 and 2 and varicella zoster virus (VZV) in CSF were negative. Serological tests were negative for *Borrelia burgdorferi*, but reactive for TBEV (IgG positive, IgM negative). The results were consistent with his vaccine status, as he had been vaccinated against TBEV at 19, 18, and 5 months prior to referral. During the next 3 days, his encephalitic symptoms progressed. The patient showed persistent fever and was then transferred to our tertiary care hospital for further treatment.

The patient had a medical history of rheumatoid arthritis, for which he was treated with prednisone (5 mg once daily) and methotrexate (20 mg once weekly). These compounds were not continued after first presentation. Of note, only the last vaccine against TBEV was administered while he was under immunosuppressive treatment. He had no significant family history, recent travel, or allergies.

On admission to our hospital, his body temperature was 38.8°C, he was disoriented and somnolent [Glasgow Coma Score (GCS) 12], and he showed neck stiffness, increased tonicity of the upper and lower extremities, increased jerks of the lower limbs, and myoclonic movements.

Repeated CSF analysis revealed a WBC count of 36/μL (99% lymphocytes) and repeated PCR tests for HSV 1, HSV 2, VZV, cytomegalovirus, enterovirus, and cryptococcal antigen remained negative. Screening for *Mycoplasma* antibodies and for vasculitis *via* multiple blood tests was negative, and a search for paraneoplastic causes by a CT scan of the chest and abdomen showed no pathologic results. Antimicrobial agents were stopped after microbiological investigations returned negative results.

**Figure 1:**
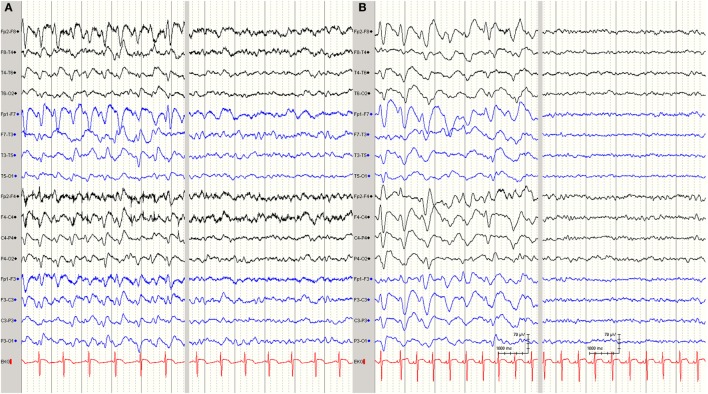
**(A)** Electroencephalogram (EEG) (bipolar display) showing epileptic activity with discharges of generalized sharp waves, alternating with phases of lower amplitude, moderately slowed activity without epileptiform signals. **(B)** Follow-up EEG under antiepileptic medication, showing broader transients in now more triphasic configuration with more phase lag, alternating with suppressed phases of a now lower amplitude.

**Figure 2:**
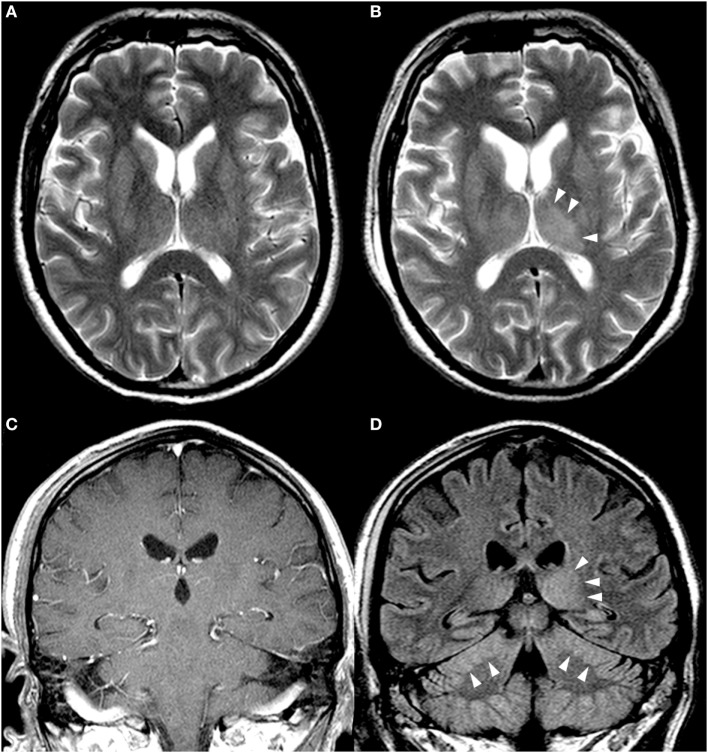
**Cerebral magnetic resonance imaging (MRI): (A,B) T2-weighted axial scans**. **(C)** T1-weighted coronal scan after intravenous application of gadolinium contrast medium. **(D)** Coronal scan with fluid attenuated inversion recovery imaging (FLAIR). **(A)** MRI on day 3. **(B–D)** MRI on day 16, showing an asymmetry with a minimal increase of signal intensity in the left thalamus **(B,D)** and in the cerebellum, with slight swelling and compression of the cerebellar sulci **(D)**. No pathological contrast enhancement was seen **(C)**.

Multiple electroencephalograms (EEGs) showed generalized slow-wave activity (Figure 1A). Within 3 days, the patient became comatose and his GCS fell to 4. He developed a syndrome of inappropriate antidiuretic hormone secretion and severe peripheral axonal neuropathy. The initially preserved oculocephalic reflex with positive doll’s eye phenomenon was soon abolished, and his GCS decreased to 3. The initial cranial magnetic resonance imaging (MRI) scan demonstrated no abnormal findings (Figure 2A) nor did the subsequent cranial CT scan on days 7 and 10. On day 9, periods of rhythmic epileptiform signal transients occurred with bifrontal localization. Antiepileptic medication was started with levetiracetam. Subsequent EEGs showed further slowing of background activity, with the appearance of triphasic signals (Figure 1B). Electroneuromyography demonstrated the absence of motor responses, even upon maximal electrical nerve stimulation, but the presence of somatosensory evoked potentials. The cranial MRI scan on day 16 showed slight hyperintensity in the region of the left lateral thalamus (Figures 2B–D).

**Figure 3:**
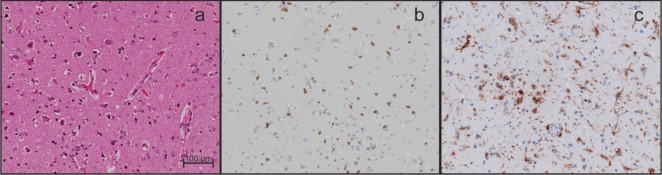
**Histopathological analyses of brain biopsy**. **(A)** Images show a mild hypercellularity. **(B)** Diffuse infiltration by T cells (CD3-positive). **(C)** Marked microglial activation (HLA-DR-positive). Magnification 200×. Scale bar corresponds to 100 µm.

An open brain biopsy on day 15 showed scant infiltration of the cerebral cortex by CD3-positive T-lymphocytes and microglial activation (Figure 3). Immunostaining for HSV 1 and HSV 2 was negative. The findings were consistent with viral encephalitis with consecutive immune response or cellular infiltration in cerebral parenchyma due to an autoimmune disease. However, neither treatment with corticosteroids nor with plasmapheresis resulted in significant improvement of brain function or peripheral nerve function.

On the basis of a high risk for persistent tetraplegia (extensive axonal peripheral motor denervation on electromyography with absence of motor responses on electrical stimulation) and for lifelong ventilator dependence and a poor cerebral prognosis, his family elected to provide comfort measures only. Four weeks after the onset of symptoms, the patient died. Shortly before the patient’s demise, IgM antibodies against West Nile virus (WNV) were detected in the serum.

## Methods and Results

West Nile virus is not endemic in Switzerland (i.e., to the best of our knowledge, no cases have ever been reported). Thus, cross-reactivity with another flavivirus was the most plausible hypothesis to explain the detection of anti-WNV-IgM in serum. Therefore, frozen samples of serum and CSF, as well as CNS tissue samples, were reanalyzed postmortem.

### Analyses of Serum Samples

Serum was reanalyzed with a flavivirus immune fluorescence assay (BIOCHIP flavivirus-mosaic 2 immunofluorescence assay; EUROIMMUN, Germany) and revealed high anti-TBEV antibody titers that cross-reacted with WNV, yellow fever virus, Japanese encephalitis virus, and dengue virus. TBEV IgG and IgM levels were retrospectively determined simultaneously in serum samples obtained on days 9 and 22 after onset of symptoms by performing an enzyme-linked immunosorbent assay (ELISA) with the Virotech Kit (FSME/TBE IgG/IgM ELISA; Sekisui Virotech, Germany). There was a 138-fold increase in anti-TBEV IgM levels and a 103-fold increase in anti-TBEV IgG levels between days 9 and 22. Avidity testing of the anti-TBEV IgG antibodies revealed highly avid anti-TBEV IgG antibodies, implying a secondary immune response. Serum obtained on day 4 was reanalyzed with real-time reverse transcriptase (RT)-PCR for WNV and TBEV; in addition, pan-flavivirus PCR was performed. The results of all assays were negative.

### Analyses of CSF Samples

Massive intrathecal IgM (92%) and IgG (73%) production could be documented in the CSF obtained on day 22 by using the scheme of Reiber and Felgenhauer. Subsequently, TBEV-specific intrathecal IgM and IgG antibody synthesis was proven by highly elevated CSF/serum antibody indices. CSF obtained on day 4 was also reanalyzed with RT-PCR for WNV and TBEV, and a pan-flavivirus PCR was performed. The results of these assays were also negative.

### Histopathology

**Figure 4:**
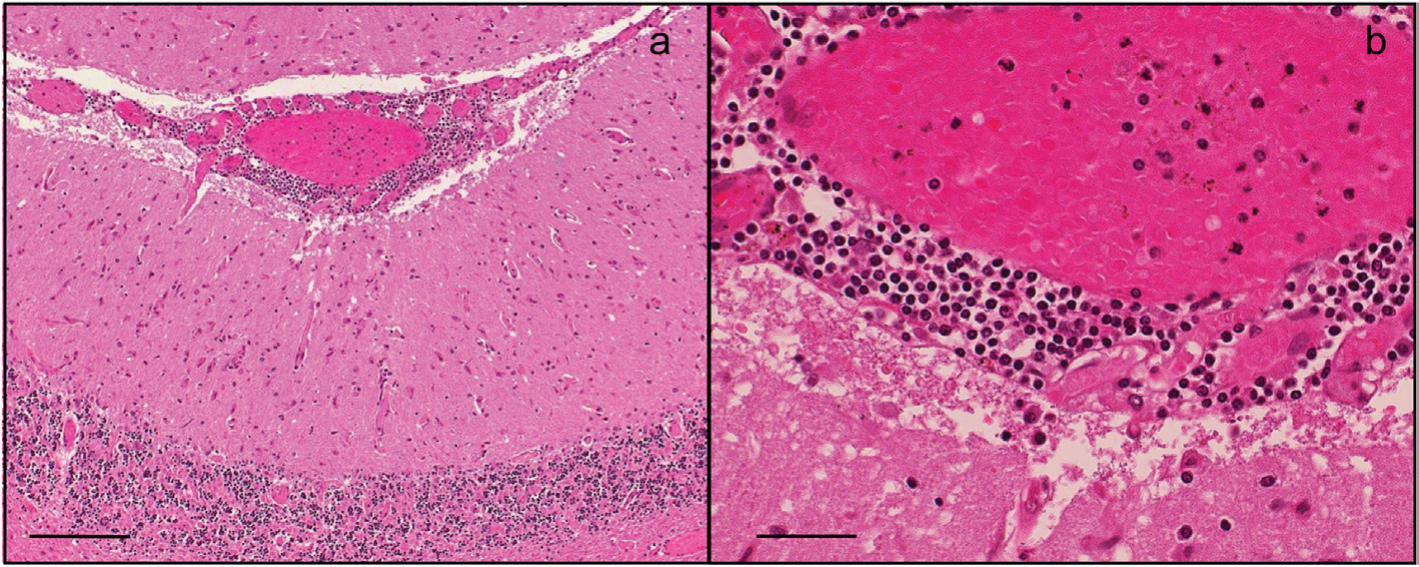
**Histopathological analyses of brain tissue at autopsy**. Hematoxylin and eosin-stained section. **(A)** Parenchymal infiltrates in a widespread distribution, multiple foci of lymphocytic meningitis. Magnification 100×. Scale bar corresponds to 200 µm. **(B)** Infiltrates in leptomeninges. Magnification 400×. Scale bar corresponds to 50 µm.

**Figure 5:**
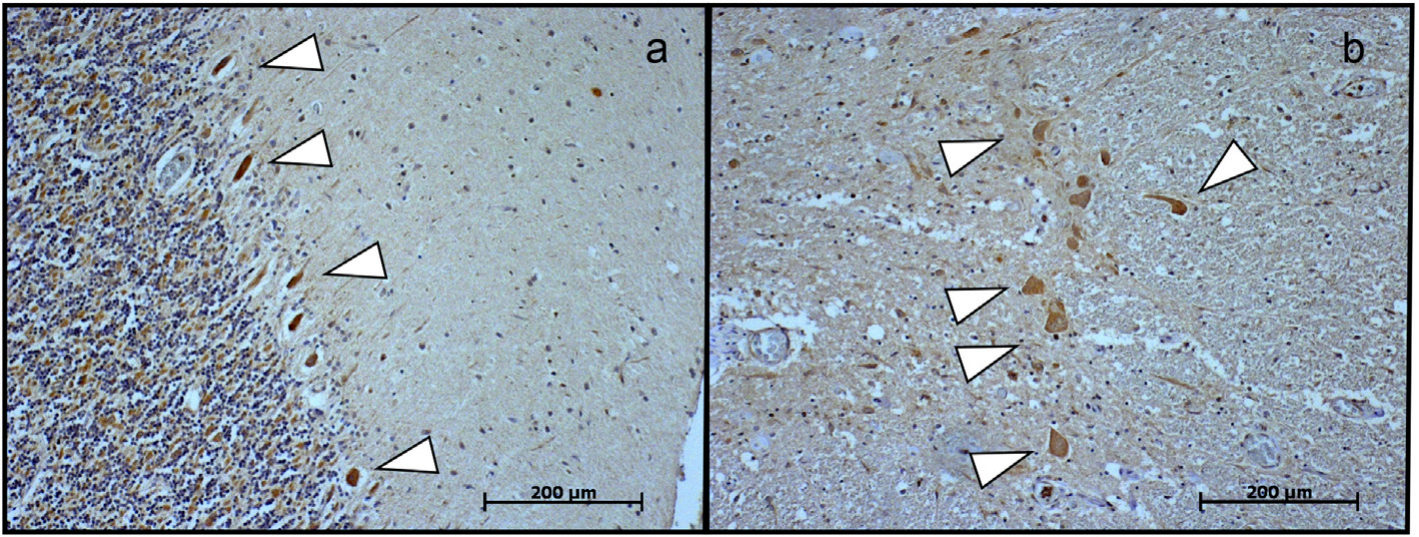
**(A)** Immunohistochemical detection of tick-borne encephalitis virus antigen in the cerebellum and **(B)** in the spinal cord revealed the presence of the virus (arrows) in Purkinje cells and in large neurons in the anterior horn. Magnification 100×. Scale bar corresponds to 200 µm.

At autopsy, the brain showed marked generalized edema. Histologically, there was lymphocytic infiltration of the hippocampi, basal ganglia brain stem, Purkinje cell layer of the cerebellar cortex, anterior horns of the spinal cord, and leptomeninges (Figure 4). Neurogenic atrophy was confirmed in the left psoas muscle. RT-PCR for the detection of TBEV was performed on postmortem samples from the cerebellum and spinal cord. High copy numbers of viral RNA were found in both samples. Immunohistochemical analysis for the demonstration of TBEV antigen was performed on tissue samples of the spinal cord and the cortical cerebellum. Paraffin sections (6 µm) were stained by using the horseradish peroxidase method (EnVision™; Dako) and a previously characterized rabbit polyclonal antibody generated against the Hochosterwitz TBEV isolate (kindly donated by Prof. Franz X. Heinz, University of Vienna, Austria) (2). Antigen retrieval was performed by using the microwave/citrate buffer method. Before mounting, slices were counterstained with hematoxylin and eosin. The presence of viral antigens was detected in the Purkinje cells of the cerebellum (Figure 5A), consistent with other confirmed cases of TBEV encephalitis (2). Immunopositive cells were also detected in sections of the spinal cord, mostly in the anterior horn (Figure 5B).

## Discussion

The final diagnosis was severe TBE with spinal motor neuron injury despite a complete course of vaccination.

An epidemiological study in Switzerland noted that 1,055 TBE cases occurred between 2005 and 2011. The average yearly incidence was 2.0/100,000 inhabitants, and 0.7% of these individuals died (3). Elaboration of the vaccination history demonstrated that in 72% of these cases, individuals were not immunized, and in 22% of cases, the immunization history was unknown. Heinz et al. (1) assessed the efficacy of vaccination coverage against the European subtype of TBE virus in Austria. The authors calculated a field effectiveness for a regularly vaccinated person at between 96 and 99%. Reports on vaccine failures in adequately immunized persons are rare (4). In Switzerland, the vaccine schedules consist of three intramuscular injections: at 0, 1–3, and then 5–12 months after the second vaccine. In European countries, there are variations in recommendations regarding the schedule of primary and booster vaccinations (5). The role of comorbidity (e.g., rheumatoid arthritis) or immunosuppressive drugs in the insufficient vaccine response remains unknown in the present case (6). Only the last vaccine was administered while the patient was under methotrexate treatment. Nonetheless, in patients with rheumatoid arthritis, the standard TBEV vaccine schedule may not confer enough immunogenicity (7).

The spectrum of clinical manifestations of TBE is broad, ranging from mild meningitis to severe encephalitis. It may be accompanied by myelitis and acute flaccid paralysis (8). In patients with encephalitis and peripheral neuropathy, the differential diagnosis of critical illness neuropathy or Guillain–Barré syndrome is often raised. By contrast, in this case, pure motor axonal neuropathy was found. This finding was due to anterior horn motoneuron damage, which is characteristic of TBE (9) and indicates TBE or vaccine failure. However, vaccine failures can be missed if antibody titers are measured only in the early phase of the disease (4, 10). The combination of a history of a completed course of vaccination, the early serology results consistent with vaccination, and the rare incidence of vaccine failures markedly delayed the diagnosis in this case. The dynamics of the antibody response during the course of disease (4, 10) and the molecular diagnostics from CNS tissue biopsies are helpful in diagnosing vaccine failure cases (11, 12). By contrast, RT-PCR in serum and CSF is typically negative when anti-TBEV antibodies are present (12).

In the present case, the vaccine-induced T-cell and antibody response failed and subsequently allowed the virus to invade the brain parenchyma and the spinal cord. The initial brain biopsy revealed a predominant T-cell-driven parenchymal infiltration. This finding is in line with other fatal TBE cases (13). We also demonstrate the presence of TBEV in the anterior horn of the spinal cord and neurogenic atrophy of the psoas muscle. The histopathological picture indicates direct motor ne uron damage by the virus and, less dominantly, indirect damage by an inflammatory response. This finding was surprising in light of previously hypothesized pathogenic concepts. Gelpi et al. demonstrated that immunologic mechanisms contribute to nerve cell destruction in human TBE (14). In addition, CD4+ T cells play an important role in the TBEV vaccine response (15). An animal infection model with lymphocytic choriomeningitis virus demonstrated that a vaccine that elicits CD4+ T cells can result in lethal immunopathology following challenge with a persistently replicating virus (16). The inflammation found in the tissue of the patient was not described to the same extent as in the animal study. The possibility of multiple different pathogenetic mechanisms in vaccination breakthrough is open to further studies.

Treatment of TBE—irrespective of vaccination status—is mainly supportive, including the use of intensive care and assisted ventilation when required (8). Because only a limited number of cases with vaccine failures are reported, it cannot be concluded that they have a more severe clinical course and a poorer prognosis. Most published vaccine failures have demonstrated an unfavorable course (17–19), but a publication bias is likely. The prognosis of TBE is determined not only by meningoencephalitic inflammation but also, as shown in this case, by spinal cord damage caused by the virus. This direct damage can result in persistent palsy of one or more limbs and even respiratory muscles. In this case, motor neuron palsy was generalized with tetraplegia and cranial nerve involvement. Complete and generalized motor neuron degeneration has no possibility of recovery and indicates a very poor prognosis indeed.

## Conclusion

This case illustrates that TBE can occur despite a full course of vaccination. The TBEV vaccine schedule may not provide enough cellular immunogenicity in immunocompromised patients. Clinicians should keep in mind that detection of specific TBEV IgG antibodies in the absence of IgM antibodies at the onset of clinical symptoms does not exclude TBE in previously vaccinated patients. Pure motor neuropathy is a characteristic finding of TBE and should raise suspicion for its diagnosis. The dynamics of the antibody response during the course of the disease and PCR from CNS samples are helpful to diagnose vaccine failures.

## Ethics Statement

Investigations or interventions were performed within routine diagnostics for this patient. As this is a case report, without experimental intervention into routine care, no formal research ethics approval was required. Written, fully informed consent for autopsy was given and recorded from the patient’s next of kin.

## Author Contributions

PS and AN were involved in the workup of the patient, planning and conducting investigations, and providing clinical care. SP and EH preformed serological and histopathological analyses, respectively, revised the manuscript, and approved the final manuscript as submitted. RA-G and DG performed RT-PCR analyses and specific immunohistopathological analyses, respectively, reviewed and revised the manuscript, and approved the final manuscript as submitted. AN interperted EEG and MRI results, revised the manuscript, and approved the final manuscript as submitted. PS and CH planned the case report, drafted the initial manuscript, reviewed and revised the manuscript, and approved the final manuscript as submitted.

## Conflict of Interest Statement

The authors declare that the research was conducted in the absence of any commercial or financial relationships that could be construed as a potential conflict of interest.
